# High-Fat Diet Aggravates the Disorder of Glucose Metabolism Caused by Chlorpyrifos Exposure in Experimental Rats

**DOI:** 10.3390/foods12040816

**Published:** 2023-02-14

**Authors:** Jinwang Li, Xiude Li, Zhihui Zhang, Weilong Cheng, Guangmin Liu, Guoping Zhao

**Affiliations:** 1 School of Food and Health, Beijing Technology and Business University, Beijing 100048, China; 2 National Center of Technology Innovation for Dairy, Huhhot 013757, China; 3 Institute of Agri-Food Processing and Nutrition, Beijing Academy of Agricultural and Forestry Sciences, Beijing 100097, China

**Keywords:** chlorpyrifos, high-fat diet, glucose metabolism, liver dysfunction, antioxidant damage

## Abstract

Epidemiological research has demonstrated that the increase in high fat consumption has promoted the morbidity of diabetes. Exposure to organophosphorus pesticides (such as chlorpyrifos) may also increase the risk of diabetes. Although chlorpyrifos is a frequently detected organophosphorus pesticide, the interaction effect between chlorpyrifos exposure and a high-fat diet on glucose metabolism is still unclear. Thus, the effects of chlorpyrifos exposure on glucose metabolism in rats eating a normal-fat diet or a high-fat diet were investigated. The results demonstrated that the glycogen content in the liver decreased and that the glucose content increased in chlorpyrifos-treated groups. Remarkably, the ATP consumption in the chlorpyrifos-treatment group was promoted in the rats eating a high-fat diet. However, chlorpyrifos treatment did not change the serum levels of insulin and glucagon. Notably, the contents of liver ALT and AST changed more significantly in the high-fat chlorpyrifos-exposed group than in the normal-fat chlorpyrifos-exposed group. Chlorpyrifos exposure caused an increase in the liver MDA level and a decrease in the enzyme activities of GSH-Px, CAT, and SOD, and the changes were more significant in the high-fat chlorpyrifos-treatment group. The results indicated that chlorpyrifos exposure led to disordered glucose metabolism in all dietary patterns as a result of antioxidant damage in the liver, in which a high-fat diet may have aggravated its toxicity.

## 1. Introduction

In recent years, a high-fat (HF) diet is becoming increasingly prevalent. This leads to an increasing incidence of obesity, with obesity rates reaching 1211 million cases in 2020 [1]. In addition to obesity, the incidence of diabetes also increased [2]. The pathophysiology of diabetes seems to be largely attributable to the excessive flux of fatty acids [3]. Obesity has been listed as an important risk factor for Type 2 diabetes mellitus and other metabolic syndromes [4,5]. According to WHO statistics, the incidence of diabetes in 2021 was 10.5%, comprising about 537 million patients worldwide. This is expected to reach 11.3% (643 million) by 2030 and 12.2% (783 million) by 2045. Diabetes is an ongoing health concern that will continue to present challenges in the future [6].

Numerous epidemiological studies have shown that toxic and harmful substances in the environment (especially organophosphorus pesticides) may play a big role in the pathogenesis of diabetes [7]. People exposed to organophosphorus pesticides (such as chlorpyrifos—CPF) usually experience an increased prevalence of diabetes and mortality [8,9]. A recent population cohort study with 6593 adult volunteers further supported this hypothesis, in which the concentrations of urinary organophosphorus pesticide metabolites showed a positive correlation with the prevalence of diabetes [10]. Organophosphorus pesticides are widely used in agricultural production worldwide. CPF is one of the most frequently used organophosphorus pesticides in many countries [11]. It is frequently detected in various food materials, including fruits, bee products, and meat (such as pesticide data in 2021 from the USA Department of Agriculture, www.ams.usda.gov/datasets/pdp, accessed on 28 December 2022) [12,13,14]. Thus, humans are exposed to CPF at high frequency, in addition to an HF dietary pattern. 

Previous reports have shown an association between CPF exposure and abnormal glucose metabolism [15]. In a cross-sectional study of 904 individuals, pancreatic dysregulation with respect to exocrine enzymes and diabesity was found in Cameroonian people who have been exposed to organophosphorus pesticides including CPF, indicating a risk of diabetes and dysregulations of insulin and glycemia [16]. It was also reported that 5 mg/kg b.w. CPF exposure led to increased fasting glucose and decreased insulin sensitivity in mice [17]. Meanwhile, CPF exposure at 5 mg/kg b.w. could impair the insulin sensitivity of mice eating both a normal-diet (NF) and an HF diet by constricting the thermogenic reaction of diet-induced brown adipose tissues [18]. CPF exposure also led to imbalanced blood glucose in different phases of the life cycle [19,20]. However, in response to the HF dietary pattern and low pesticide exposure dose (closer to the actual human exposure dose), the effect of CPF on glucose metabolism and the degree to which an HF diet would interfere with the toxic effect of CPF have yet to be clearly elucidated. A toxic mechanism that cannot be ignored is the oxidative damage and antioxidant damage CPF causes in mammals [21] since a higher plasma level of aspartate aminotransferase (AST) was observed in diabetic mice exposed to CPF at doses of 1 and 2 mg/kg b.w. [22].

In order to further understand the health risks resulting from pesticide exposure and the current HF dietary pattern, this research aimed to explore the interaction between an HF diet and CPF on glucose metabolism by comparing the effects of CPF exposure on rats under the feeding mode of an HF and NF diet.

## 2. Materials and Methods

### 2.1. Materials and Chemicals

Wistar rats (male of 7 weeks) were provided by Weitong Lihua Laboratory Animal Technology Co. (Beijing, China). The rats’ NF and HF diets, which were largely powered by fat (40%) were supported by Shanghai Slack Laboratory Animal Co. (Shanghai, China). The main components of the NF and HF diets were prepared according to previous reports [23]. CPF with purity over 97% was provided by Huaxia Regent Company (Chengdu, China). All other chemicals used were analytical grade.

### 2.2. Design of the Experiment

First, CPF was dissolved in dimethyl sulfoxide (DMSO). Then, the CPF solution was prepared through dilution into 0.9% saline containing 0.5% Tween-20 before it was given to rats (the final DMSO concentration was 0.1%). A total of 24 Wistar rats (252.9 ± 19.3 g) were randomly and equally separated into 4 groups. The settings of the four groups were the NF-CON group (treated with NF diet and solvent of CPF), the NF-CPF group (treated with NF diet and 3.0 mg/kg b.w. of CPF), the HF-CON group (treated with HF diet and solvent of CPF), and the HF-CPF group (treated with HF diet and 3.0 mg/kg b.w. of CPF). The concentration of CPF 3.0 mg/kg b.w. corresponded to the dose of 1/50 of LD50 [24,25]. All of the rats were treated daily for 20 weeks via gavage. 

Once the experiment time reached 20 weeks, all rats in the 4 groups were anesthetized immediately for collection of blood from orbits. Then, the rats were sacrificed to collect and weigh the liver tissue. Once the stewing time of blood reached 2 h under 25 °C, the blood was centrifuged at 5000× *g* for 20 min under 4 °C. Then, the upper serum was removed to a new tube and stored. Meanwhile, the organ relative weight was calculated using the following formula (Equation (1)).

Organ relative weight (%) =100 × organ weight (g)/final body weight (g) (1)

All of the rat samples were stored under −80 °C until further detection. The experimental design was approved by the China Agricultural University Laboratory Animal Welfare and Animal Experimental Ethical Inspection Committee (CAU20170108-3). The rats from all four groups were treated humanely and measures were taken to prevent suffering.

### 2.3. Glucose Homeostasis Measurements

At the start of the 20th week, the rats fasted for 12 h. Then, an oral glucose tolerance test was performed via gavage of 2 g/kg b.w. of glucose. After that, one drop of blood was post-injected from the rat tail, and the blood glucose values were detected at 0, 15, 30, 60, 90, 120, and 180 min using a glucometer (Roche, ACCU-CHEK Active, Germany).

At the end of the 20th week, rats fasted for 6 h, and ITT was evaluated after an intraperitoneal injection of 0.75 U/kg b.w. insulin. Then, the blood glucose concentrations were detected at 0, 15, 30, 60, 120 min, and 180 min. 

The area under the curve from 0 to 180 min (AUC0–180) was calculated according to the trapezoidal rule formula following (Equation (2)).

AUC = 1/2 × (t_15_ − t_0_) × (C_0_ + C_15_) + 1/2 × (t_30_ − t_15_) × (C_15_ + C_30_) … (2)

where t is the time point, and C is the blood glucose value.

### 2.4. Detection of ALT, AST, and ALP Concentration in Serum

The indicators reflecting the capacity of liver function, including serum alanine aminotransferase (ALT), aspartate aminotransferase (AST), and alkaline phosphatase (ALP), were determined after the CPF treatment. Concentrations of ALT and AST were measured by the Beijing Sino-UK Institute of Biological Technology (Beijing, China) via radioimmunoassay.

### 2.5. Antioxidant Function Determination of Liver

The content of malondialdehyde (MDA) in liver tissues was assessed using an MDA commercial kit (Nanjing Jiancheng Bioengineering Institute, Nanjing, China) following the manufacturer’s instructions. The activity of superoxide dismutase (SOD), catalase (CAT), and glutathione peroxidase (GSH-Px) were detected using commercial assay kits (Nanjing Jiancheng Bioengineering Institute, Nanjing, China) following the manufacturer’s instructions.

### 2.6. Detection of Glycogen and Glucose Content in Liver

The content of liver glycogen was determined according to the instructions included in the commercial kit (Nanjing Jiancheng Bioengineering Institute, Nanjing, Jiangsu, China). The content of glucose in the rat liver was detected using an oxidase assay kit following the manufacturer’s instructions (Beijing Puli Gene Technology, Beijing, China). The level of liver ATP was determined using an ATP detection kit (Beyotime, Beijing, China).

### 2.7. Determination of Insulin and Glucagon Concentration in Serum

The concentration of insulin and glucagon in rat serum after the CPF intervention was determined using a multiplexing kit according to the manufacturer’s protocol (Millipore, Milliplex MAP Rat Metabolic Hormone Magnetic Bead 13-Plex Panel, Burlington, MA, USA).

### 2.8. Statistical Analysis

The data were analyzed via SPSS (Version 23, IBM Corp., Armonk, NY, USA) and presented in the form of means ± standard deviation. A statistical comparison was performed by one-way ANOVA followed by Dunn’s post-test for multiple comparisons. The differences were considered significant at *p* < 0.05.

## 3. Results

### 3.1. HF Diet CPF Did Not Affect Liver Weight

**Figure 1:**
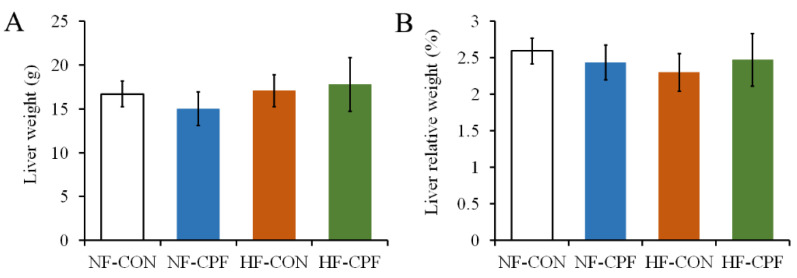
Effects of CPF on liver weight (**A**) and relative weight (to the whole bodyweight, (**B**)) of rats treated with 3.0 mg/kg b.w. CPF under the NF diet and HF diet. CPF—Chlorpyrifos. NF-CON—fed a normal-fat diet and vehicle. NF-CPF—fed a normal-fat diet and chlorpyrifos. HF-CON—fed a high-fat diet and vehicle. HF-CPF—fed high-fat diet and chlorpyrifos.

The liver is the main detoxifying organ for pesticides [11]. It is also important for fat metabolism. As shown in Figure 1A, the liver weight of rats in the NF-CPF group was slightly reduced, but there was no significant difference from that of the NF-CON group (*p* > 0.05). There was no difference in the liver weight between the HF-CON group and the NF-CON group; the same was true for the HF-CPF group (*p* > 0.05). 

These results indicated that 3.0 mg/kg b.w. CPF exposure did not show a negative effect on liver weight in response to the NF dietary pattern or the HF dietary pattern. Similarly, the organ’s relative weight was unaltered by CPF exposure. Meanwhile, the HF dietary pattern did not aggravate the toxicity of CPF on liver weight (Figure 1B, *p* > 0.05).

### 3.2. HF Diet Promoted a More Severe Glucose Intolerance for CPF Treatment

**Figure 2:**
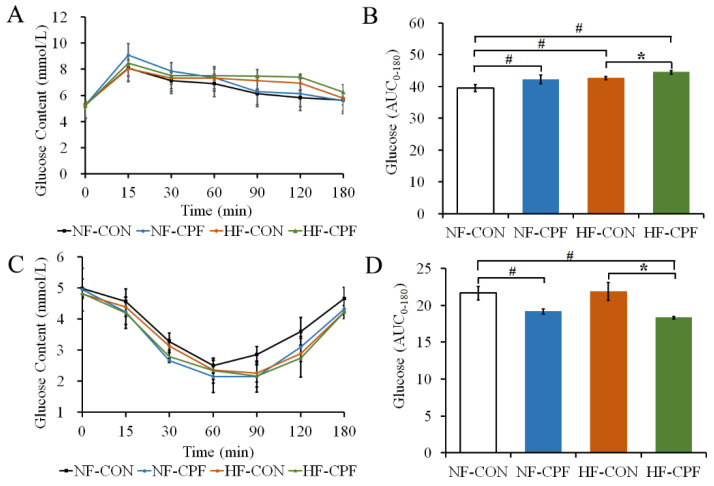
Effects of CPF on rat glucose tolerance and insulin tolerance in response to NF diet and HF diet. (**A**) The blood glucose content of rats after different points were gavaged with glucose. (**B**) Total glucose content of the area under the glucose curve during the whole testing period in (**A**). (**C**) The blood glucose content of rats after insulin injection at different points. (**D**) Total glucose content of the area under the glucose curve during the whole testing period in (**C**). CPF—Chlorpyrifos. NF-CON—fed a normal-fat diet and vehicle. NF-CPF—fed a normal-fat diet and chlorpyrifos. HF-CON—fed a high-fat diet and vehicle. HF-CPF—fed a high-fat diet and chlorpyrifos. The symbols * and # indicate a significant difference between the covered groups at *p* < 0.05.

In order to clarify the effects of the HF diet and CPF exposure on glucose metabolism, the glucose homeostasis and insulin sensitivity of rats were determined (Figure 2). The results showed that the fasting blood glucose of rats in CPF-exposure groups (both NF diet and HF diet) demonstrated no significant difference compared with the control group, indicating that CPF treatment did not affect the fasting blood glucose of rats with NF or HF diet status (Figure 2A,C, *p* > 0.05). CPF treatment disturbed the insulin sensitivity of rats with NF diet status since the area below the blood glucose line in the CPF-treatment group within 180 min of insulin injection was significantly lower than that in the control group (Figure 2D, *p* > 0.05). The HF diet did not increase the area below the blood glucose line for 180 min of insulin injection (*p* > 0.05). The area under the curve in the HF-CPF-treatment group significantly decreased compared with that in the HF-CON group (*p* < 0.05). The area under the curve in the HF-CPF group was even slightly lower than that in the NF-CON group. These results indicated that CPF (3 mg/kg b.w.) could negatively affect insulin tolerance in rats and that the HF diet possibly promoted its toxicity.

Interestingly, within 180 min of oral glucose administration, the area below the blood glucose line in the NF-CPF treated group was significantly higher than in the NF-CON group (Figure 2B, *p* < 0.05). The HF diet also led to a higher area below the blood glucose line than the NF diet. Additionally, the area in the HF-CPF group was promoted compared with HF-CON and HF-CPF. Thus, CPF treatment paired with an HF diet aggravated glucose intolerance in rats.

### 3.3. HF Diet and CPF Co-Treatment Led to the Disorder of Glucose Metabolism

**Figure 3:**
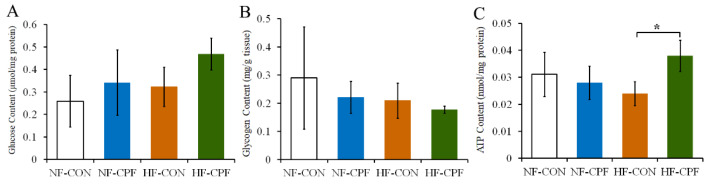
Effects of CPF on liver glucose metabolism of rats under NF diet and HF diet. (**A**) Glucose content. (**B**) Glycogen content. (**C**) ATP content. CPF—Chlorpyrifos. ATP—Adenosine triphosphate. NF-CON—fed a normal-fat diet and vehicle. NF-CPF—fed a normal-fat diet and chlorpyrifos. HF-CON—fed a high-fat diet and vehicle. HF-CPF—fed a high-fat diet and chlorpyrifos. The symbol * indicates a significant difference between the covered groups at *p* < 0.05.

Under the NF dietary pattern, CPF treatment increased the glucose content in the liver of rats compared with the control group, while the liver glycogen content in the CPF treatment group decreased compared with the control group. The above indicators were aggravated in the CPF HF diet co-treatment group. The glycogen content of rats in the HF-CPF group was lower than that in the NF-CPF group, and the glucose content was higher in the HF-CPF group than that of rats treated with CPF alone (Figure 3A,B). These results indicated that CPF treatment resulted in a tendency toward hepatic glucose metabolism disorder in rats. Furthermore, ATP concentration was detected in the liver of CPF-treated rats. As shown in Figure 3C, CPF treatment decreased the ATP concentration in the rat liver. The concentration of ATP in the HF-CON group also decreased compared with that in the NF-CON group. However, the liver ATP concentration in the CPF-treatment group increased in response to the HF diet. Meanwhile, the difference was significant compared with the HF-CON group (Figure 3C, *p* < 0.05).

### 3.4. CPF Treatment Did Not Destroy the Function of Islet Cells in Rats under NF or HF Diet

**Figure 4:**
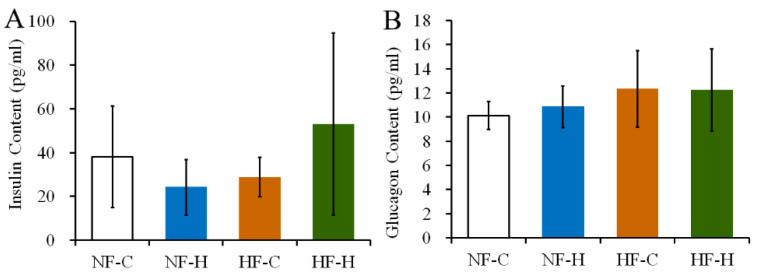
Effects of CPF on serum contents of insulin (**A**) and glucagon (**B**) in rats treated under the NF diet and HF diet. CPF—Chlorpyrifos. NF-CON—fed a normal-fat diet and vehicle. NF-CPF—fed a normal-fat diet and chlorpyrifos. HF-CON—fed a high-fat diet and vehicle. HF-CPF—fed a high-fat diet and chlorpyrifos. There are no significant differences between all of the experimental groups at *p* < 0.05.

Under the NF dietary pattern, the serum insulin concentration in rats after the CPF treatment was lower than that of the control group, but there was no significant difference. The HF diet did not alter the concentration of serum insulin in rats of the control or CPF groups (Figure 4A, *p* > 0.05). The CPF treatment also did not change the serum glucagon level in rats fed with the NF diet and HF diet (Figure 4B, *p* > 0.05). These results indicated that CPF did not damage the blood glucose regulation function of islet cells in rats under the NF or HF diet.

### 3.5. CPF Treatment Damaged Liver Function of Rats under HF Diet

**Figure 5:**
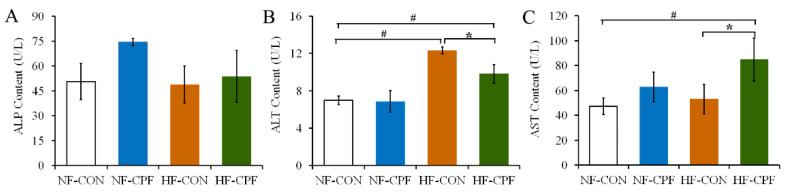
Effects of CPF on liver function of the rat under the NF diet and HF diet. (**A**) ALP content. (**B**) ALT content. (**C**) AST content. CPF—Chlorpyrifos. NF-CON—fed a normal-fat diet and vehicle. NF-CPF—fed a normal-fat diet and chlorpyrifos. HF-CON—fed a high-fat diet and vehicle. HF-CPF—fed a high-fat diet and chlorpyrifos. The symbol * and # indicates a significant difference between the covered groups at *p* < 0.05.

Under the NF dietary pattern, the serum ALP and AST concentrations in CPF-treated rats showed an increasing trend compared with the NF-CON group, but there was no significant difference (Figure 5A,C, *p* > 0.05). There were no significant changes in ALT concentration between the CPF treatment group and the control group. In response to the HF dietary pattern, the serum ALT concentration significantly increased (Figure 5B, *p* < 0.05). The serum concentrations of ALT and AST in the CPF-treated group were significantly higher than those in the control group under the HF diet (Figure 5B,C, *p* < 0.05), indicating that the HF diet promoted CPF-induced liver function damage.

### 3.6. CPF and HF Diet Co-Treatment Damaged the Antioxidant Function of Liver

**Figure 6:**
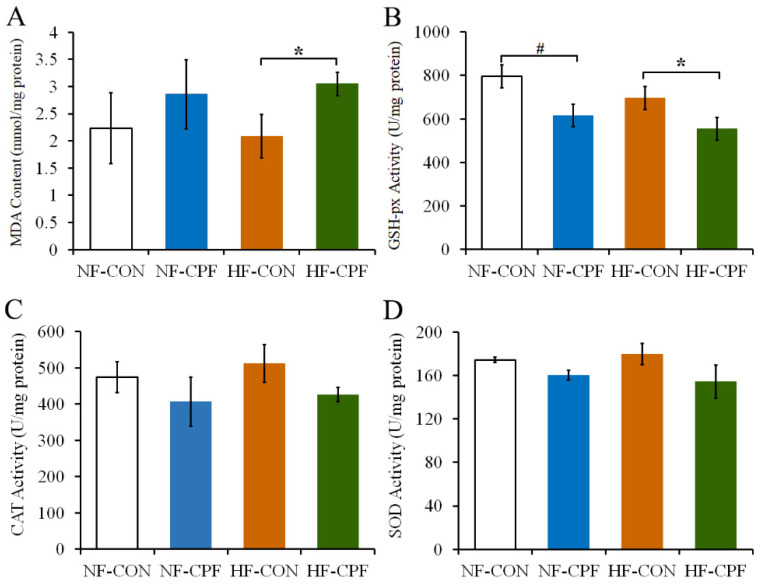
Effects of CPF on the antioxidant system of the liver. (**A**) MDA content. (**B**) GSH-px activity. (**C**) CAT activity. (**D**) SOD activity. CPF—Chlorpyrifos. NF-CON—fed a normal-fat diet and vehicle. NF-CPF—fed a normal-fat diet and chlorpyrifos. HF-CON—fed a high-fat diet and vehicle. HF-CPF—fed a high-fat diet and chlorpyrifos. The symbol * and # indicates a significant difference between the covered groups at *p* < 0.05.

As the results show, the content of liver MDA in the CPF-treatment group was higher than that in the control group. Furthermore, the HF diet aggravated this phenomenon, which manifested as significantly increased MDA activity in the HF and CPF co-treatment groups compared with the HF-CON group (Figure 6A, *p* < 0.05). The increase in MDA activity indicated an occurrence of oxidative damage under the CPF treatment. In addition, CPF could decrease the main antioxidant enzyme activity of the liver (such as SOD, CAT, and GSH-Px) in response to the NF and HF diets (Figure 6B–D). Notably, the GSH-Px activity in NF-CPF and HF-CPF groups decreased remarkably compared with their corresponding control group, respectively (Figure 6B, *p* < 0.05), indicating a decrease in the antioxidant capacity in the rat liver.

## 4. Discussion

It has been reported that CPF exposure could cause abnormal glucose metabolism in the body, leading to the occurrence and development of other diseases and, thus, presenting a health threat [17]. This research investigated the effects of the HF diet on CPF-induced glucose metabolism disorders and antioxidant function in rats. The results of insulin tolerance and glucose tolerance showed that CPF exposure did not affect the function of islets under the NF diet or an HF diet. However, the HF diet possibly promoted CPF-induced negative effects on glycemic function, leading to disordered glucose metabolism in the rat liver. 

Once the body’s glucose content has been maintained at a high level for a long time, it struggles to return to normal, and the body may have a tendency toward hyperglycemia [26]. The glycemic and hypoglycemic functions of the body were directly determined by glucagon and insulin in the blood [27]. Therefore, the serum levels of these two hormones were measured in rats, and the results showed that CPF exposure did not change the insulin or glucagon levels in rats regardless of whether they ate an NF or HF diet. Therefore, it was hypothesized that CPF might interfere with glucose metabolism through other pathways under the HF dietary pattern. In this research, the CPF exposure group also showed a decreasing trend in glycogen and an increasing trend in glucose content. Population blood samples collected from 300 participants in Cameroon and Pakistan demonstrated the presence of organophosphorus pesticide residues, including CPF, malathion, and parathion [28]. CPF was detected in samples from both Cameroonian and Pakistani samples. Significantly elevated body mass index, insulin, blood glucose, and dyslipidemia were noted in groups that were chronically exposed to organophosphorus pesticides; dysregulated liver function was also noted, regardless of gender and age. The findings reported by Javeres et al. as well as our results indicated that dysregulations related to glucose metabolism were attributed to chronic exposure to organophosphorus pesticides. Researchers found that prolonged exposure to malathion results in hyperglycemia by stimulating the glycogenolysis and gluconeogenesis reaction in hepatic cells. The possible underlying mechanism was thought to be an increased activity of glycogen phosphorylase and phosphoenolpyruvate carboxykinase induced by pesticide exposure as the activities of these two enzymes were generally higher in diabetic patients [21,29,30]. However, CPF exposure did not induce excessively higher blood glucose in response to the NF or the HF diet in this study. In addition, there were no significant differences in insulin content or glycogen content in CPF-treated groups. Thus, the fundamental link between CPF exposure and glucose metabolism has yet to be clarified. 

A study investigating the effects of pesticide exposure (diazinon) and the HF diet on insulin resistance showed that the MDA level was remarkably increased in groups treated with single diazinon and diazinon together with HF. A similar result was also found for an oxidative stress biomarker, reactive oxygen species [31]. Thus, one mechanism that might play an important role in the induction of abnormal blood glucose might be oxidative stress-related damage. The impaired antioxidant function is an important manifestation of oxidative stress and is considered an important component of the toxic mechanisms induced by several organophosphorus pesticides [32,33,34]. In the present study, the MDA level in the rat liver was also increased, indicating oxidative damage caused by CPF exposure. Treatment by 4.75 mg/kg b.w. CPF exhibited a similar phenomenon in the increase in the MDA level in rat serum [35]. In addition, some studies have reported the antioxidant imbalance effect of different organophosphorus pesticides in vivo and in vitro models [36,37]. Toxic damage to the rat liver under CPF exposure was also found in this study, manifesting as increased AST content. Similarly, another study concerning the effect of CPF on glucose metabolism implied that CPF treatment led to liver damage and immuno-inflammatory responses [22]. The oxidative damage caused by CPF was also witnessed in other organisms. For example, increased MDA content and disordered metabolites in zebrafish liver were found after CPF treatment at 30, 100, and 300 µg/L. Notably, those metabolic changes included changes in the glucose and lipid metabolism pathways [38]. Interestingly, when exposed at high doses, the organ most affected by CPF-induced hyperglycemia might be the pancreas islet or other hormone-secreting organs, which exhibit remarkable pesticide toxicity [39]. However, relatively low-dose exposure to CPF might target the liver and lead to metabolic toxicity. Ismail et al. also reported that assessment of liver and kidney functions was a sound practice for evaluating the organism injury following chronic, repeated exposure to CPF [40].

CPF is highly fat-soluble and easily stored in fat and other lipophilic tissues. The liver is an important organ for lipid metabolism, and it is also an important organ for the metabolism and detoxification of pesticides [41]. Thus, CPF is easily stored in the liver. Meanwhile, the liver is important for glucose homeostasis and storage of antioxidation-related enzymes (such as GSH-Px, CAT, and SOD, which were detected in this study). The above facts made it vulnerable to CPF-induced glucose metabolic disorder [42]. In this research, we found that hepatic glycogen content showed a decreasing trend and glucose content showed an increasing trend after CPF exposure, while the ATP concentration increased more significantly in response to the HF diet and CPF exposure. The result showed that the glucose metabolism disruption was more serious for the increased ATP concentration. This might be because the glycogenolysis of the liver was activated, and the activated glycogenolysis promoted glucose release or hyperglycemia in Type 2 diabetics [43]. Interestingly, a high-fat diet seemed to exhibit negative effects in aggravating the severity of glucose metabolic disorders. The negative effect of the HF diet on the disturbance of glucose metabolism caused by pesticides seemed to have been identified and recognized by some researchers [44]. The fat-solubilizing property of CPF appeared to promote the disruption of glucose metabolism induced by it.

It was suggested that the glycogen decomposition and gluconeogenesis increased as an antioxidative detoxification mechanism induced by organophosphorus pesticides themselves [29]. Meanwhile, more and more researchers found that antioxidants need glucose to counteract free radicals. Glucose is used as a substrate of glucose-6-phosphate, which plays an important role in the transition of oxidized glutathione to a reduced state [45]. Glutathione and GSH-Px are both involved in the detoxification process of CPF in the liver. In this study, the glucose contents increased, while the activities of GSH-Px decreased in both NF- and HF-CPF-treated groups. Similar to the report of Basiri et al. [29], it is possible that glycogenolysis and gluconeogenesis processes in the liver were stimulated to offer glucose and provide a source of energy for cellular antioxidants to counteract free radicals.

## 5. Conclusions

This research indicated that relatively low-dose CPF chronic exposure led to glucose metabolism disorders both under the HF and NF diet. Furthermore, the mechanism of CPF-induced glucose metabolism disorders was possibly not a result of damage to the function of the pancreas islet; it may have occurred via antioxidant damage in the rat liver. The HF dietary pattern promoted the above disruption of glucose metabolism-related indicators, with a similar extent of antioxidant damage. Thus, the HF diet might compound the health risk caused by CPF exposure.

## Data Availability

Data presented in this research are available on request from the corresponding author.
